# *CORL* Expression in the *Drosophila* Central Nervous System Is Regulated by Stage Specific Interactions of Intertwined Activators and Repressors

**DOI:** 10.1534/g3.118.200282

**Published:** 2018-05-30

**Authors:** Nancy L. Tran, Norma T. Takaesu, Elizabeth F. Cornell, Stuart J. Newfeld

**Affiliations:** School of Life Sciences, Arizona State University, Tempe AZ 85287-4501

**Keywords:** Fussel/SKOR, twin of eyeless, Drifter, dILP2, pars intercerebralis

## Abstract

CORL proteins (SKOR in mice and Fussel in humans) are a subfamily of central nervous system (CNS) specific proteins related to Sno/Ski oncogenes. Their developmental and homeostatic roles are largely unknown. We previously showed that Drosophila *CORL* (*dCORL*; fussel in Flybase) functions between the Activin receptor Baboon and Ecdysone Receptor-B1 (EcR-B1) activation in mushroom body neurons of third instar larval brains. To better understand *dCORL* regulation and function we generated a series of reporter genes. We examined the embryonic and larval CNS and found that *dCORL* is regulated by stage specific interactions between intertwined activators and repressors spanning numerous reporters. The reporter AH.lacZ, which contains sequences 7-11kb upstream of *dCORL* exon1, reflects *dCORL* brain expression at all stages. Surprisingly, AH.lacZ was not detected in EcR-B1 expressing mushroom body neurons. In larvae AH.lacZ is coexpressed with Elav and the transcription factor Drifter in dILP2 insulin producing cells of the pars intercerebralis. The presence of dCORL in insulin producing cells suggests that dCORL functions non-autonomously in the regulation of EcR-B1 mushroom body activation via the modulation of insulin signaling. Overall, the high level of sequence conservation seen in all CORL/SKOR/Fussel family members and their common CNS specificity suggest that similarly complex regulation and a potential function in insulin signaling are associated with SKOR/Fussel proteins in mammals.

Secreted Transforming Growth Factor-β (TGF-β) proteins belong to either the Decapentaplegic (Dpp)/Bone Morphogenetic Protein (BMP) or the TGF-β/Activin subfamilies. Members of both subfamilies perform a myriad of tasks during embryonic development and adult homeostasis. Mutations disrupting TGF-β pathways in adults can lead to problems such as cancer, fibrosis, cardiovascular disease and inflammation. One mechanism of regulating TGF-β functions is via proteins that interact with Smad signal transducers in TGF-β responsive cells. One group of Smad interacting proteins are the Sno/Ski family of proto-oncogenes. Sno/Ski proteins have a multi-faceted relationship with Smads that typically depends upon the cellular context. Mammalian SnoN was initially shown in tumor models to be an obligate antagonist of TGF-β signaling. Subsequently developmental studies in flies and mice revealed other interactions. For example in flies, dSno (referred to as Snoo in Flybase) acts as a pathway switch. It facilitates TGF-β/Activin signaling via a molecular mechanism that simultaneously antagonizes Dpp/BMP signaling (Takaesu *et al.* 2006). Available mouse data suggests an analogous switch function but in reverse - SnoN supports signaling by Dpp/BMP while antagonizing TGF-β/Activin. Consistent with this dual role, human SnoN can function as an oncogene and a tumor suppressor gene (Zhu and Luo 2012). *dSno* was also shown to have a role as an antagonist of Wnt signal transduction during wing development (Quijano *et al.* 2010).

mCORL1 was first identified as a co-repressor for the homeodomain transcription factor Lbx1. Analyses of embryonic development showed that it is expressed only in dorsal interneurons of the cerebellum (Mizuhara *et al.* 2005). Developmental studies of mCORL2 showed that this gene is expressed only in Purkinje cells of the cerebellum (Minaki *et al.* 2008). These genes were recently renamed mSKOR1 and mSKOR2. Loss of function studies of mSKOR2 show that it is necessary for proper Purkinje cell differentiation during development. This is thought to occur via the activation of Sonic hedgehog signaling in Purkinje cells (Miyata *et al.* 2010; Wang *et al.* 2011). This result was supported by a knockout study revealing that mSKOR2 promotes the differentiation of Purkinje cells by inhibiting interneuron fate (Nakatani *et al.* 2014). No knockout studies of mSKOR1 have been reported. mSKOR1 is primarily, though not exclusively, expressed in the cerebellum of adults while mSKOR2 expression is restricted to the cerebellum in adults (Yue *et al.* 2014).

There are two human Fussel proteins named for their chromosomal location. Fussel15 is homologous to mSKOR1 and Fussel18 is homologous to mSKOR2. Fussel15 expression is conserved with mSKOR1 in the adult cerebellum. There are also low levels of transcription in the lung and small intestine. Like its homolog mSKOR2, Fussel18 is expressed uniquely in the adult cerebellum (Fagerberg *et al.* 2014). Both Fussel proteins bind Smads non-specifically in transfected melanoma cells. Luciferase assays in these cells suggest Fussel18 inhibits while Fussel15 has no effect on TGF-β signaling (Arndt *et al.* 2005; Arndt *et al.* 2007). Genome-wide association studies have linked mutations in three human genes (Fussel15/hSKOR1, MEIS1 and BTBD9) to two ataxias in American, European and Chinese populations - Restless Leg Syndrome and Periodic Leg Movement Syndrome (Kemlink *et al.* 2009; Moore *et al.* 2014; Li *et al.* 2017). Both ataxias are thought to result from dysfunction in the cerebellum, as that is the site of movement in the brain. No syndromes are yet associated with mutations in Fussel18/hSKOR2.

In the only study of a *dCORL* mutation, one aspect of its CNS specific expression was shown to function downstream of the Myoglianin receptor Baboon in the activation of EcR-B1 in the larval mushroom body. Note the identity of the ligand was unknown at the time. Biochemical studies of mCORL1 revealed a specific interaction with mSmad3. Taken together these data suggested that dCORL is a Smad-interacting protein that facilitates Myoglianin signal transduction in mushroom body neurons (Takaesu *et al.* 2012). There is a study employing ectopic *dCORL* expression in wings that suggests a role antagonizing BMP signaling (Fischer *et al.* 2012). One of the human genes associated with Restless Leg Syndrome has been validated in flies (BTBD9). Mutations in the BTBD9 counterpart CG1826 disrupt sleep and the data suggests this is due to dysregulation of brain dopamine levels (Freeman *et al.* 2012). Association of the fly versions of Fussel15/hSKOR1 (dCORL) or MEIS1 (homothorax) with aberrant behaviors has not yet been reported.

Here we describe a regulation/expression study of *dCORL* employing reporter genes that revealed stage specific interactions between activators and repressors spread across a 12kb region. We also identify insulin producing neurons of the larval brain as a site of *dCORL* expression. We propose that similarly complex transcriptional regulation and a potential function in insulin signaling are associated with SKOR/Fussel proteins in mammals.

## Materials and Methods

### dCORL Reporter Gene Construction

A set of eleven *dCORL* reporter genes was constructed in pHZR (Gindhart *et al.* 1995). These span 38,225bp of genomic DNA and were generated in two phases. The first phase of four reporters covered the non-coding DNA in a region of 23,446bp surrounding the *dCORL* ORF (Figure 2A). There were two small gaps: 54bp of *dCORL* intron3 and 514bp between the distal end of Int2.lacZ and the proximal end of 5′lacZ. These gaps amount to only 1.5% of the non-coding sequences in this region. Reporter generation was initially guided by the structure of *dCORL* cDNA LD43973 (fuss-RC in Flybase; a detailed exon/intron map of transcripts fuss-RB and fuss-RC is in Takaesu *et al.* (2012). 3′.lacZ is 4238bp and begins at the BspHI site (957,996bp; Ref Seq NC_004353.4) within the divergently transcribed CG32016. It ends at the BlpI site within *dCORL* exon5 (962,234bp). Int4.lacZ is 1224bp and begins at the MfeI site (962,262bp) within *dCORL* exon5. It ends at the BsrBI site (963,486bp) within *dCORL* exon4. Int2.lacZ is 3763bp and begins at the *Bam*HI site (965,026bp) within *dCORL* exon3. It ends at the *Spe*I site just proximal to *dCORL* exon2. 5′.lacZ is 6252bp and begins at the AclI site (969,303bp) just distal to *dCORL* exon2. It ends at the NgoMIV site (975,555bp) within the first exon of the divergently transcribed non-coding RNA *sphinx*. Failure to detect any expression in embryos or third instar larval brains with this first set of reporters suggested that LD43973 exons1 and 2 (fuss-RC in Flybase) were artifacts and did not represent a true *dCORL* transcript. We then found the actual start of *dCORL* on the distal side of *sphinx* via 5′RACE (fuss-RB in Flybase; Takaesu *et al.* 2012). This discovery guided construction of the second phase of seven reporters covering the 14,809bp intergenic region (Figure 2B). This set begins proximal to *sphinx* and ends within the divergent *toy* exon1. KB.lacZ is 5775bp and it begins at a Kpn site proximal to the first exon of *sphinx* (974,998bp). It overlaps 5′.lacZ by 378bp and contains *sphinx*, *dCORL* exon1 of fuss-RB and 2773bp upstream ending at BlpI (980,762bp). BA.lacZ is 4670bp and starts at BlpI. It ends at AvrII (985,432bp) midway between *dCORL* and *toy*. BB.lacZ contains the proximal 3491bp of BA.lacZ, starting at BlpI and ending at *Bgl*II (984,253bp). BgK.lacZ is 3408bp starting at *Bgl*II and continuing past the end of BA.lacZ to *Kpn*I (987,661bp) 2064bp upstream of *toy* exon1. BpK.lacZ is 6899bp encompassing all of BB.lacZ and BgK.lacZ starting at BlpI and ending at *Kpn*I. AH.lacZ is 4365bp starting within BgK.lacZ and BpK.lacZ at AvrII and ending at *Hin*dIII (989,797bp) within *toy* exon1. KH.lacZ is 2136bp and starts at *Kpn*I (987,661bp). It contains the distal region of AH.lacZ, including 2064bp immediately upstream of *toy* exon1 and 72bp of *toy* exon1. It ends at *Hin*dIII (989,797bp) within *toy* exon1. Two independent insertions were tested for each reporter.

### Whole Mount RNA and Antibody Detection in Embryos and Larvae

For RNA *in situ* and antibody staining 0-12 hr embryo collections at 25° were aged 12 hr (final age 12-24 hr) for analysis. Larvae that have stopped wandering but not yet begun pupariation (prior to anterior spiracle eversion - equivalent to 122 hr at 25° for *yw*) were picked individually, sorted by sex and their CNS dissected in groups of 9-12. Antibody and RNA double labeling for the light microscope was as described (Takaesu *et al.* 2012). For 3-color confocal detection of antibodies, tissues were fixed in 4% formaldehyde, rinsed and stored in methanol until needed. Primary antibodies in figures were: rabbit and rat α-lacZ (Organon Teknika, Durham; MBL, Nagoya), mouse α-EcR-B1 (DSHB AD4.4), guinea pig α-Toy (gift of Uwe Walldorf, Univ. Saarland), mouse α-En (DSHB 4D9), guinea pig α-Eve (gift of John Reinitz, Univ. Chicago), mouse α-Fas2 (DSHB 1D4), rat α-Elav (DSHB 7E8A10), mouse-α Repo (DSHB 8D12), guinea pig α-Drifter (gift of Makoto Sato, Univ. Kanazawa) and rat α-dILP2 (gift of Pierre Leopold, Univ. Nice). See Table S1 for additional antibodies. Secondary antibodies were: goat α-mouse, α-rabbit, α-guinea pig or α-rat Alexa Fluor 488, 546 and 633 (Molecular Probes). RNA *in situ* probes were transcribed from a full-length clone of fuss-RB generated in our lab as described (Takaesu *et al.* 2012). For 2-color detection of RNA and antibodies the *dCORL* α-sense probe was detected using the Molecular Probes Tyramide Signal Amplification kit with Alexa Fluor 488 labeled tyramide. The primary antibody for labeled RNA probes was sheep α-dig (Roche, Penzburg) and the secondary was donkey α-sheep-HRP (Novex, Fredrick). Tissues were imaged on a Leica confocal with slices every 2µm.

### Bioinformatics

Genomic coordinates derive from the *D. melanogaster* chromosome 4 complete sequence (Ref Seq NC_004353.4). The 16,960bp region from *Sma*I (974,049bp) within 5′.lacZ proximal to *sphinx* through *Bam*HI (991,004bp) within *toy* intron1 was aligned across the 12 Drosophila reference genomes with Evoprinter (Yavatkar *et al.* 2008). This span includes the 14,809bp contained in the second set of seven reporter genes. Conserved sequences in the alignment were scanned with *cis*-Decoder for consensus binding sites (Brody *et al.* 2007).

### Data Availability

Strains are available upon request. The authors affirm that all data necessary for confirming the conclusions of the article are present within the article, figures, and tables. Supplemental material available at Figshare: https://doi.org/10.25387/g3.6356759.

## Results

### dCORL embryonic expression is regulated by intertwined activators and repressors

**Figure 1:**
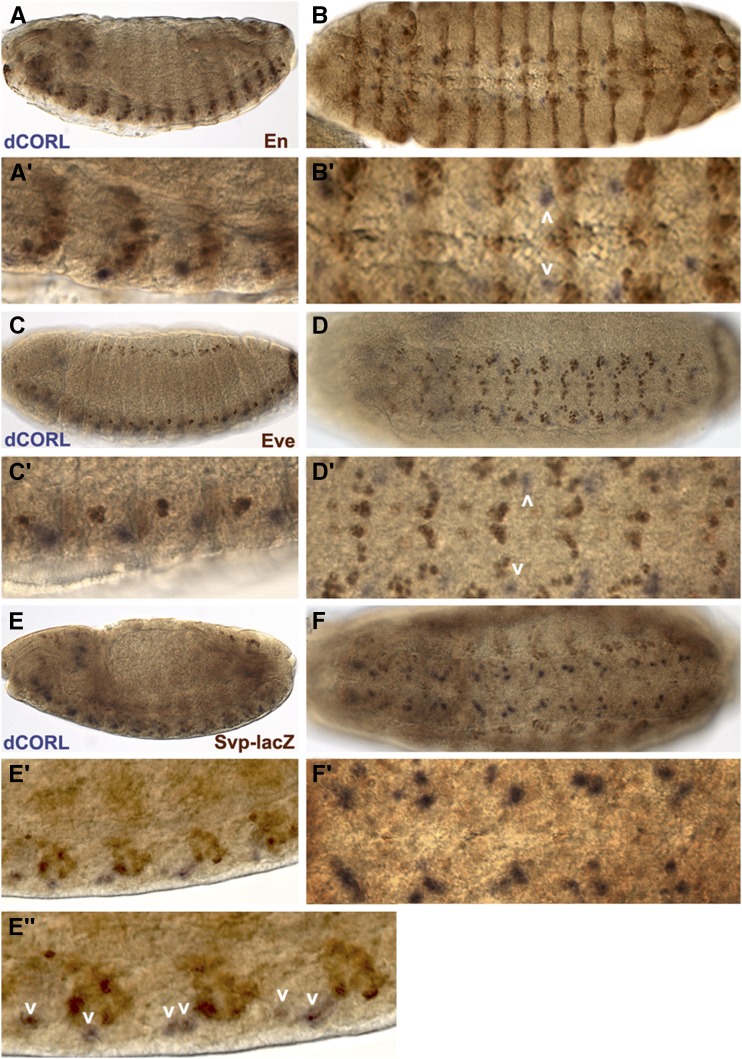
*dCORL* is present in embryonic ventral cord cells that are neither En, Eve nor Svp positive. *dCORL* RNA in stage 14 wild type embryos with antibodies as indicated. Left column is lateral and right column is ventral view at higher magnification. A,B) En (brown) and *dCORL* (purple) are shown in A) medial and B) apical view. En is present in ventral cord cells and ectodermal stripes in both views. Employing the nomenclature of Doe (1992), En is found in rows NB-6 and NB-7 at the posterior end of each segment. *dCORL* is located apical and anterior to each row of En (white arrowheads in B’). C,D) Apical views of Eve (brown) and *dCORL* (purple). Eve is observed in ventral cord cells and groups of ectodermal cells lateral to the ventral cord. In the ventral cord, Eve marks ganglion mother cells aligned along the anterior-posterior axis in the medial region of each segment. *dCORL* is located apical and lateral to Eve ventral cord cells. On the anterior-posterior axis *dCORL* cells (white arrowheads in D’) are between Eve ganglion mother cells derived from NB4-2 and NB6-2. E,F) Stage 15 medial views of Svp-lacZ (brown) and *dCORL* (purple). Svp-lacZ is visible in ventral cord cells and in groups of ectodermal cells lateral to the ventral cord. In the ventral cord, Svp-lacZ marks many neuroblasts and ganglion mother cells in each segment. *dCORL* cells are located anterior and apical to a subset of Svp cells in a stereotypic position that corresponds to the axis of neuroblast to ganglion mother cell division for NB5-4, NB5-5 or NB5-6 (white arrowheads in E”).

To identify *dCORL* neurons in the embryonic ventral cord we examined stage 14 embryos double labeled for *dCORL* RNA and three markers of ventral cord neuroblasts (Engrailed, Even-Skipped and Seven-up; Doe 1992). Engrailed (En) marks the two posterior-most rows of neuroblasts (NBs; rows 6 and 7) in each segment and the anterior-most neuroblast (NB1-1) such that En neuroblasts align into a band stretching across the ventral cord at each segment boundary. *dCORL* cells are located apical and anterior to the band of En neuroblasts (Figure 1A,B). Even-skipped (Eve) marks the first ganglion mother cell derived from NB1-1, NB4-2 and NB6-2. Subsequently, the NB1-1 and NB4-2 ganglion mother cells become aCC/pCC and RP2/RP2sib neurons while NB6-2 ganglion mother cell becomes a pair of interneurons in the U/CQ group (Doe 1992). *dCORL* cells are located apical and lateral to all Eve cells and fall between Eve cells derived from NB4-2 and NB6-2 on the anterior-posterior axis (Figure 1C,D). Seven-up (Svp) is expressed in 24 of the 29 neuroblasts in each segment as well as in the second ganglion mother cell derived from NB5-2 and NB7-4 (Doe 1992). *dCORL* cells are located apical and lateral to Svp cells in a position that corresponds to the stereotypic axis of neuroblast to ganglion mother cell asymmetric division (45° offset from the embryo surface; Doe 1992). *dCORL* cells appear to be generated from the anterior-most Svp neuroblast in a group of five to six neuroblasts occupying the same lateral optical plane (Figure 1E,F). Based on the location of *dCORL* cells between Eve NB4-2 and NB6-2 ganglion mother cells and the arrangement of the visible Svp neuroblasts we speculate that *dCORL* cells are generated by NB4-3 or its peripheral-posterior neighbor NB5-4.

**Figure 2:**
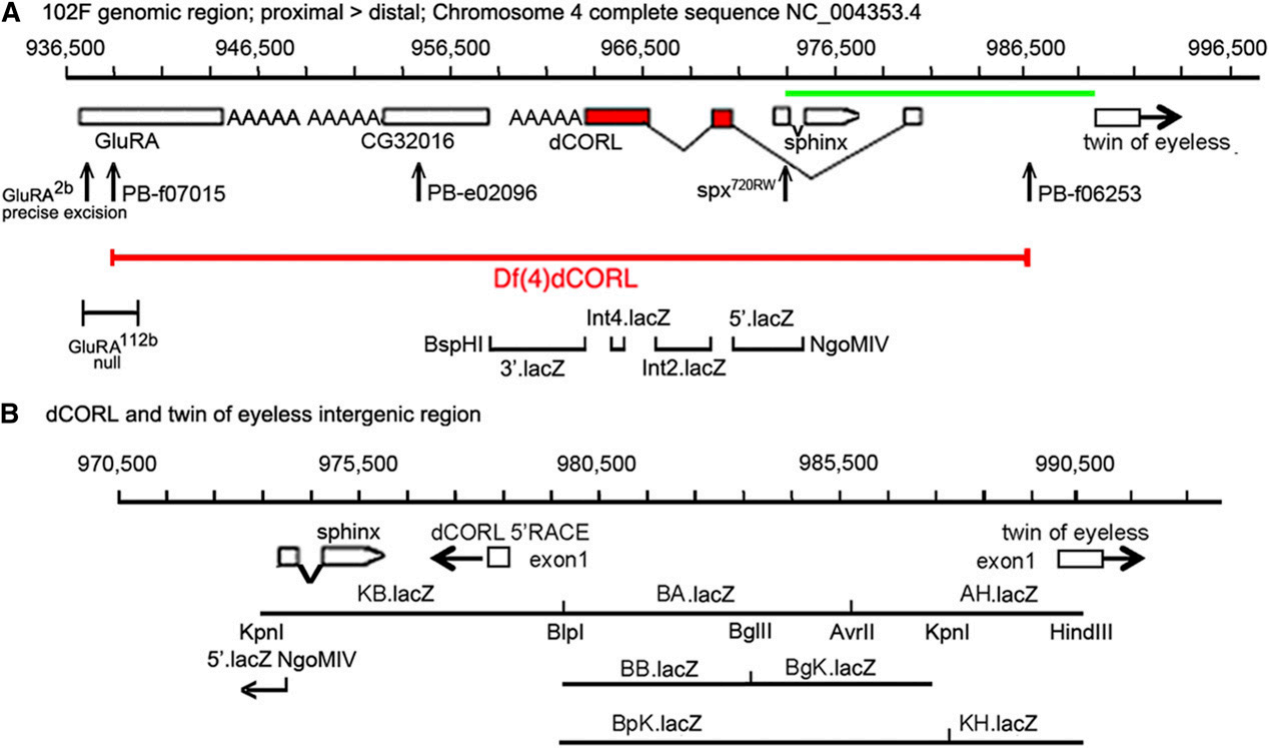
Eleven reporter genes covering *dCORL*. A) Genomic map of 60kb around *dCORL* from 936,500bp to 996,500bp on chromosome 4. Boxes representing genes are enlarged at this scale for visibility but are appropriately placed and proportional to each other. *dCORL* spans 962,341bp to 978,489bp (Ref Seq NC_004353.4) with exons from the longest of three known *dCORL* transcripts indicated (transcript-RB in Flybase). Red boxes contain the ORF and V-shaped lines indicate splicing (small introns3&4 not shown). *dCORL* transcription is anti-parallel. The *Df(4)dCORL* deletion eliminates *dCORL*, *Glu-RA* (936,819bp), *CG32016* (951,542bp) and the non-coding RNA *sphinx* (975,168bp transcribed in parallel). The parallel transcribed *toy* (exon1 989,725-990,502bp) is located distal to *Df(4)dCORL* (Takaesu *et al.* 2012). The insertions *PB^f07015^* (936,128bp) and *PB^f06253^* (979,771bp) are FRT-containing transgenes employed to generate *Df(4)dCORL* via introchromosomal recombination. *PB^e02096^* disrupts *CG32016*. *Glu-RA^2b^* is a precise excision reverting to wild type and *Glu-RA^112b^* is a deletion that acts as a null. *Sphinx^720RW^* is an allele engineered to interrupt splicing. Four nuclear-lacZ reporters were created in the HZR vector (high levels of expression yield nuclear and cytoplasmic staining). They begin proximally at the BspHI site in *CG32016* (957,996bp) and end distally at the NgoMIV site (975,555bp) in the first exon of *sphinx*. They are shown with the location of their genomic DNA inserts in relation to the *dCORL* ORF from proximal to distal: 3′.lacZ, Int4.lacZ, Int2.lacZ and 5′.lacZ. The green line spanning the intergenic region between *dCORL* and *toy* is expanded below. B) Genomic map of 20kb from 970,500bp to 990,500bp. Boxes representing genes are shown to scale as well as appropriately placed and proportional to each other. *dCORL* and *toy* exon1 are shown (divergently transcribed) as well as *sphinx*. Seven HZR lacZ reporters with the locations of their genomic DNA are indicated. Restrictions sites that mark reporter ends are shown, as is the overlap of the distal end of 5′.lacZ (NgoMIV 975,555bp) and the proximal end of KB.lacZ (Kpn 947,998bp). The seven reporters of the second set span a region (14,809bp) that is larger than the formal intergenic region (11,236bp), so as to leave no gap with the first reporter set. The group of seven reporters begins proximal to *sphinx*, include *dCORL* exon1 and extend distally into *toy* exon1. See Materials and Methods for details.

To allow for confocal imaging, we generated eleven *dCORL* reporter genes in two phases. First, we created a set of four non-overlapping reporters that covered: 1) the region between the proximal gene *CG32016* and the 3′ end of *dCORL*, 2) two of the three *dCORL* introns within the ORF and 3) the region between *dCORL* exon2 where the ORF begins and *sphinx* (Figure 2A). These did not display any expression in embryos or third instar larval brains. Second, we created a set of seven overlapping reporters covering a 14809bp intergenic region beginning at the proximal side of *sphinx* and ending distally within the divergently transcribed *twin of eyeless* (*toy)* exon1 (Figure 2B).

**Figure 3:**
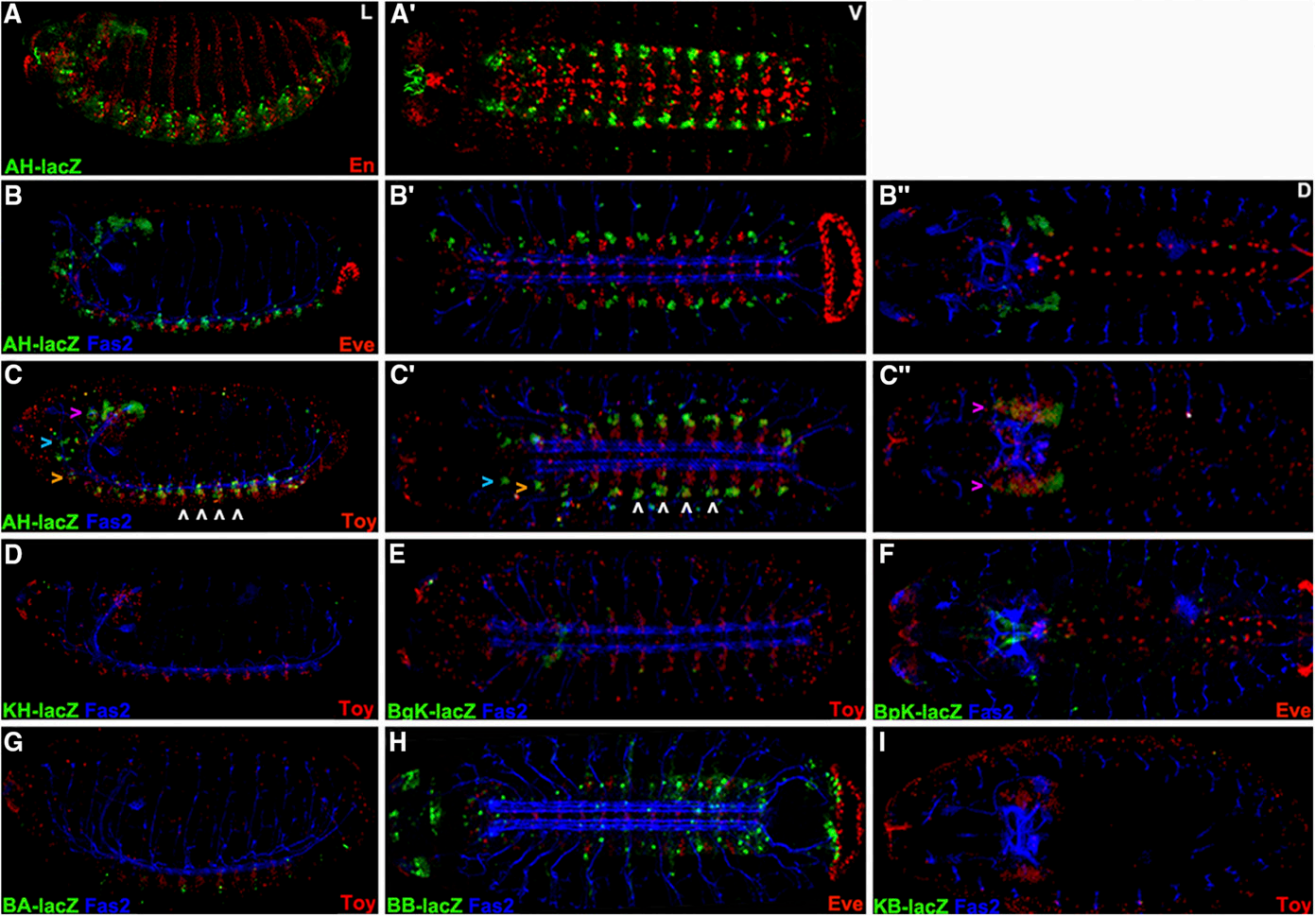
*dCORL* embryonic reporter expression reveals intertwined activators and repressors. Lateral (left), ventral (center) and dorsal (right) views of stage 16 embryos displaying Fas2 as a neural marker (blue), lacZ (green) and Eve or En or Toy (red). Rows A,B) AH.lacZ is not expressed with En or Eve (no yellow cells). Row C) Lateral and ventral views show that AH.lacZ is expressed in the ventral cord in a small group of bilaterally symmetrical cells per hemisegment at the lateral edge. Toy is also bilaterally symmetrical in the ventral cord and expressed in rows of nine-ten cells aligned from medial to lateral. In each segment AH.lacZ is expressed in several of the lateral-most Toy cells - seen as yellow in C,C’ (examples indicated with white arrowheads). Lateral view shows that AH.lacZ is also present in the trito- deuto- and protocerebrum of the brain (from ventral to dorsal - orange, aqua and purple arrowheads respectively). Protocerebral lacZ expression overflows the nuclei and is visible in cell bodies. Toy expression is also present in the protocerebrum. While coexpression in the protocerebrum appears present in the dorsal view (C” - yellow cells indicated with purple arrowheads), the lateral view (C) shows that protocerebral Toy cells do not coexpress AH.lacZ. Protocerebral Toy cells are instead uniformly ventral to AH.lacZ cells in this region (details in Figure 4). D-I) Five intergenic reporters display no expression (KB.lacZ, BA.lacZ, BgK.lacZ, BpK.lacZ and KH.lacZ). BB.lacZ expression is seen in 1-2 cells per segment in the ventral cord that likely correspond to *dCORL* as they do not overlap with Toy.

We labeled stage 16-17 embryos carrying the second set of seven reporter genes to display: 1) lacZ to detect reporter expression, 2) Fas2 to mark a distinctive subset of neural membranes in the brain and ventral cord and 3) Eve or En or Toy. From these studies we noted that AH.lacZ expression in the ventral cord does not overlap with Eve or En (Figure 3A,B) but does coexpress with the most lateral Toy cells (Figure 3C). This is logical since AH.lacZ contains sequences immediately upstream of the *toy* transcription start and ends within *toy* exon1. Surprisingly KH.lacZ that contains the distal half of AH.lacZ including *toy* exon1 has no expression (Figure 3D). BgK.lacZ, BpK.lacZ and BA.lacZ also show no expression (Figure 3E,F,G). BB.lacZ starts 2273bp upstream of *dCORL* exon1 and its expression appears similar to *dCORL* RNA in the ventral cord (compare Figure 3H with Figure 1F’). KB.lacZ includes *dCORL* exon1 and the 2273bp immediately upstream but has no expression (Figure 3I).

The intergenic activators in BB.lacZ that drive *dCORL* ventral cord expression are also fully contained in BA.lacZ and BpK.lacZ that show no expression. This indicates the presence of *dCORL* ventral cord repressors and activators in the intergenic region. Also, the activators in AH.lacZ that drive Toy ventral cord expression are not present in the subset of AH.lacZ that is contained in KH.lacZ. The remainder of AH.lacZ is contained in BgK.lacZ that also has no expression. This indicates the presence of activators and repressors of Toy ventral cord expression in the intergenic region as well.

**Figure 4:**
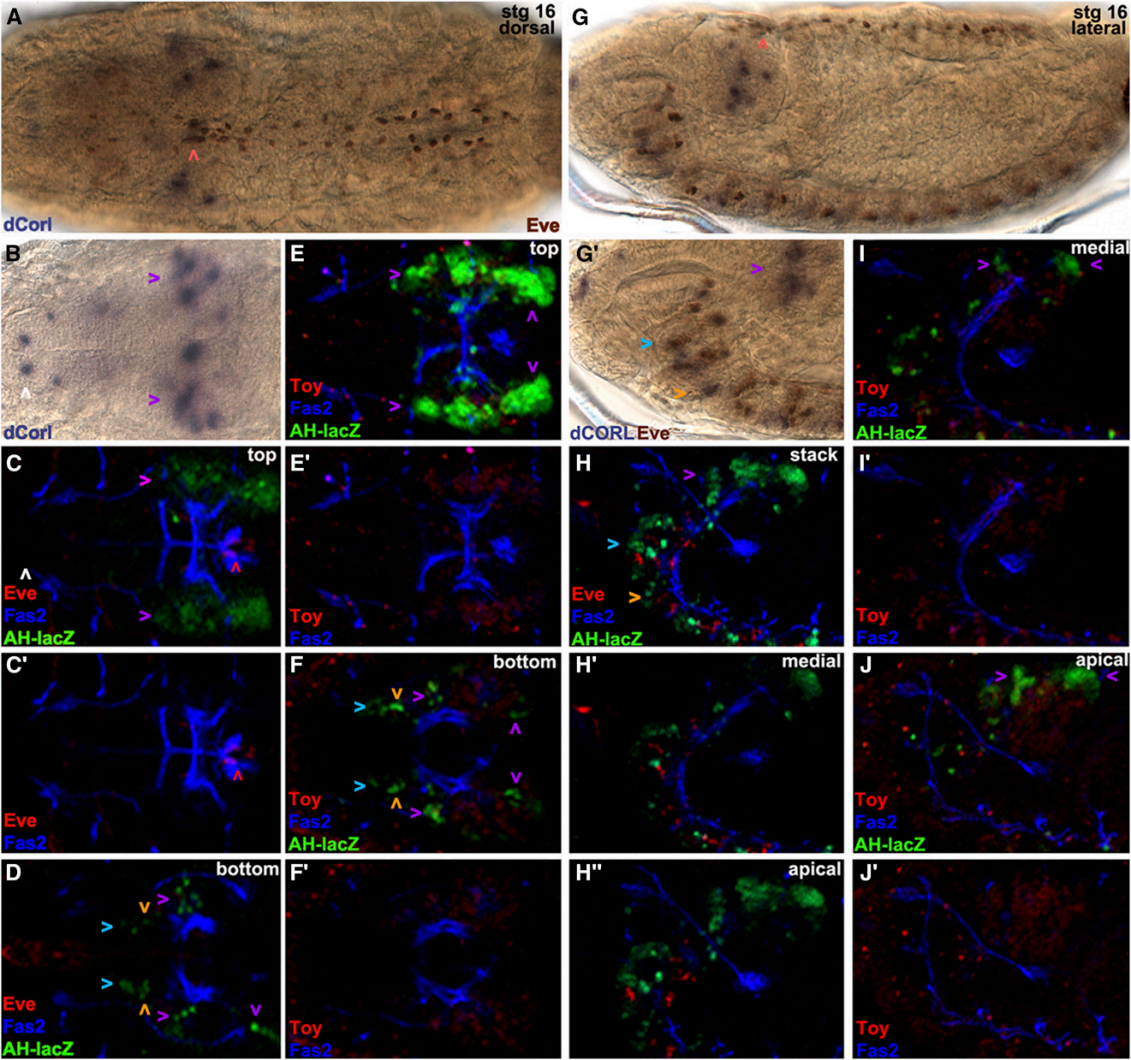
AH.lacZ embryonic brain expression reflects *dCORL* and not Toy. Stage 16 embryos. Left half dorsal views and right half lateral views. A) Eve (brown) and *dCORL* RNA (purple). Eve is present along the aorta in two anterior-posterior rows each composed of 2 pericardial cells per segment. Where the aorta squeezes between the brain lobes Eve cells are irregularly arranged (red arrowhead). *dCORL* medial brain expression appears associated with Eve at this location. B) *dCORL* RNA is located in groups of cells laterally in the brain (protoerebrum – purple arrowheads) and in a medial group of cells. *dCORL* is also found in pairs of cells at several locations along the pharynx (example shown with white arrowhead). C, C’) Top half of slices of the brain revealing Eve (red), Fas2 (blue) and AH.lacZ (green) or just Eve and Fas2. Strong protocerebral AH.lacZ expression approximating *dCORL* RNA is visible (purple arrowheads; lacZ expression overflows the nuclei and is visible in cell bodies). The accurate but incomplete reflection of *dCORL* expression of AH.lacZ is shown by the lack of expression near the irregularly spaced Eve cells (red arrowhead) and lack of expression near the pharynx (white arrowhead). D) Bottom half of slices from the same embryo displaying ventral expression in the trito-, deuto and protocerebrum (from ventral to dorsal orange, aqua and purple arrowheads, respectively). E) Top half of slices of the brain revealing Toy (red), Fas2 (blue) and AH.lacZ (green) or just Toy and Fas2. Strong protocerebral AH.lacZ expression (purple arrowheads) appears to overlay Toy expression dorsally as well as to the anterior and posterior. F) Bottom half of slices from the same embryo displaying AH.lacZ ventral expression in the protocerebrum (purple arrowheads) anterior and posterior to Toy as well as in the trito- and deutocerebrum (orange and aqua arrowheads). G) Eve (brown) and *dCORL* (purple). Eve is adjacent to *dCORL* where the aorta squeezes between the brain lobes (red arrowhead). Close-up view of the ventral brain region of the same embryo revealing *dCORL* expression in the protocerebrum (purple arrowhead). dCORL expression in the trito- and deutocerebrum (orange and aqua arrowheads) is associated with Eve. H) Complete stack, medial half of slices and apical slices from the quarter of the brain closest to the viewer revealing Eve (red), Fas2 (blue) and AH.lacZ (green). AH.lacZ trito- deuto- and protocerebral expression (orange, aqua and purple arrowheads) is visible with the former two associated with Eve. I,J) Medial half of slices and apical slices from the quarter of the brain closest to the viewer revealing Toy (red), Fas2 (blue) and AH.lacZ (green) or just Toy and Fas2. Both views show that AH.lacZ protocerebral expression (purple, arrowheads) is present anterior and posterior to Toy. Taken together (E,F,I,J) show the envelopment of protocerebral Toy expression on its dorsal, anterior and posterior sides by AH.lacZ.

In the embryonic brain, AH.lacZ expression is prominent (Figure 3B,C). Examining this in detail indicates that AH.lacZ is accurate where present, but it is not an exhaustive surrogate for *dCORL* brain transcription. Two aspects are absent: in the medial brain dorsally near Eve in the aorta (Figure 4A) and anterior to the brain along the pharynx (Figure 4B). AH.lacZ in dorsal view, when displayed with Fas2 and Eve, even after separation into top half (dorsal) and bottom half (ventral) of optical sections is not visible medially or along the pharynx (Figure 4C,D).

Replacing Eve with Toy and generating images of the top and bottom half of sections shows that AH.lacZ brain expression does not overlap with Toy. In the dorsal top view (Figure 4E), AH.lacZ is widespread and strong (overflowing nuclei and filling the cell bodies) while surrounding Toy on its anterior, dorsal and posterior sides. In the ventral bottom view (Figure 4F), Toy remains visible but only the anterior- and posterior-most “Toy surrounding” AH.lacZ cells are visible. No coexpressing cells are visible in either view indicating that AH.lacZ reflects only *dCORL* in the brain. The ventral bottom view also shows AH.lacZ expression in the trito- and deutocerebral regions of the brain that corresponds to *dCORL* RNA (visible in Figure 4G).

Examination of lateral views of the brain displaying AH.lacZ, Fas2 and Eve as either a full stack, medial half of slices or apical slices from the quarter of the embryo closest to the reader (Figure 4H) confirms the distinct aspects of *dCORL* expression revealed by the top and bottom halves of the dorsal view. Replacing Eve with Toy in the medial and apical views (Figure 4I,J) shows that the surrounding of Toy by *dCORL* includes the medial and apical sides, leading to the appearance of a “helmet” of *dCORL* surrounding a “head” of Toy. *dCORL* presence in the trito-and deutocerebrum, as seen with Eve, is confirmed.

At this time the identity of *dCORL* expressing cells in the embryo is not known. What we have learned is: 1) that AH.lacZ reflects *dCORL* in the brain and a small subset of Toy in the ventral cord, 2) embryonic activators and repressors of *dCORL* are spread throughout the 12kb intergenic region and 3) *dCORL* activators and repressors are intertwined.

### dCORL larval brain expression is present in all dILP2 expressing cells

**Figure 5:**
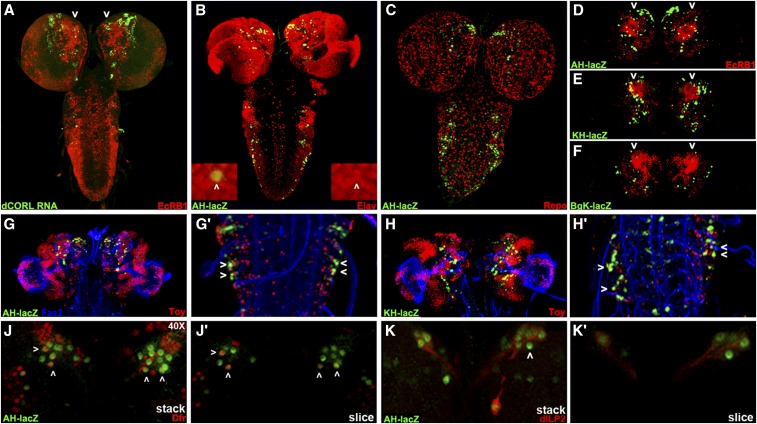
*dCORL* larval brain expression is present in all dILP2 insulin producing cells. Dorsal view of larval brains with anterior up. A) *dCORL* RNA (green) with EcR-B1 (red) in wild type are not coexpressed in the mushroom body (white arrowheads). B) AH.lacZ brain displaying lacZ (green) and Elav (red). Coexpression of AH.lacZ and Elav in neurons is visible. The left inset shows a high magnification view in two colors and the right inset the same image in red only. The white arrowhead points to a coexpressing cell. C) AH.lacZ brain displaying lacZ (green) and Repo (red). No coexpression is noted (red and green cells are adjacent). D,E,F) AH.lacZ, KH.lacZ and BgK.lacZ central brains displaying lacZ (green) and EcR-B1 (red). Each reporter reflects subsets of *dCORL* RNA expression but none is coexpressed with EcR-B1 in the mushroom body (white arrowheads). G,H) AH.lacZ and KH.lacZ brains displaying lacZ (green), Fas2 (blue) and Toy (red). Close-up views of the ventral cord show that AH.lacZ and KH.lacZ are expressed in the lateral-most Toy cells in each row (examples shown with white arrowheads). J) AH.lacZ (green) and Drifter (red) with coexpression in a subset of neurons (white arrowheads). K) AH.lacZ (green) and dILP2 (red) with coexpression in all dILP2 insulin producing cells of the pars intercerebralis - seven cells with green nuclei and red cytoplasm on the right side, the left side is slightly rolled with three cells behind the four that are visible. A single AH.lacZ cell not expressing dILP2 is visible medial to the co-expressing cells in the right hemisphere (white arrowhead). Axons from the coexpressing cells in both hemispheres extend medially and connect with a coexpressing cell outside the pars intercerebralis (right hemisphere).

To determine if *dCORL* functions autonomously or non-autonomously in the activation of EcR-B1 in the mushroom body we labeled larval brains just prior to pupariation (the stage we reported in Takaesu *et al.* 2012; larvae were no longer moving but their cuticle was white and still pliant) for *dCORL* RNA and EcR-B1 (Figure 5A). Surprisingly, we found no coexpression at this stage. Instead *dCORL* expressing cells surrounding EcR-B1 cells. This indicated to us that *dCORL* acted non-autonomously. We attempted to identify *dCORL* cells in the larval brain.

We employed AH.lacZ initially, as this reporter had the most robust embryonic expression. After side-by-side comparisons suggested that AH.lacZ larval expression depicted a significant fraction of *dCORL* transcription in the brain (Figure 5A,D), we began triple labeling studies employing Fas2 as a marker of a distinctive subset of neural membranes. We tried neurosecretory cell markers (*e.g.*, bursicon and dimmed) as well as mushroom body markers (*e.g.*, trio and dachshund). We did not find any coexpression at this stage and then examined the other reporter genes with these markers. Table S1 shows 11 antibodies that did not coexpress with any of our *dCORL* reporters.

One antibody that is coexpressed with AH.lacZ detects Elav (an RNA binding protein found in the nuclei of mature neurons; Figure 5B). Another antibody that does not coexpress recognizes Repo (a transcription factor found in the nuclei of mature glia; Figure 5C). We also examined the full complement of eleven reporter genes and found three of them displayed brain expression (AH.lacZ, KH.lacZ, and BgK.lacZ; Figure 5D-F). These three appeared to reflect overlapping subsets of *dCORL* brain expression and, like AH.lacZ and *dCORL* RNA, none were expressed in EcR-B1 neurons in the mushroom body.

The reporter experiments led to several interesting observations regarding *dCORL* larval activators and repressors in the brain and ventral cord as well as the relative location of larval and embryonic activators and repressors. First, KH.lacZ contains the distal half of AH.lacZ (see Figure 2B) ending in *toy* exon1 and it has robust larval brain expression that looks like AH.lacZ and *dCORL* RNA. The similarity of AH.lacZ and KH.lacZ indicates that a majority of *dCORL* larval brain activators reside in the distal-most part of the intergenic region immediately upstream of *toy* exon1. Second, AH.lacZ and KH.lacZ larval brain similarity is distinct from their relationship in the embryonic brain where AH.lacZ has robust expression and KH.lacZ has none. Thus repressors in KH.lacZ of AH.lacZ embryonic brain activators do not also repress larval brain activators. Third, there is no larval expression for KB.lacZ, BA.lacZ and BB.lacZ that cover the proximal half of the intergenic region including *dCORL* exon1. Fourth, BB.lacZ shows embryonic *dCORL* ventral cord expression but not larval ventral cord expression. These observations show that distinct activator/repressor combinations exist for embryonic and larval stages and that their respective activators and repressors are intertwined.

Triple labeling studies with Toy in larvae (Figure 5G,H) were consistent with two aspects of the embryonic studies. First, none of the *dCORL* reporters with larval brain expression also reflected Toy. There are incidental exceptions when one of the few reporter driven lacZ nuclei (green) is immediately above or below one of the many Toy nuclei (red) in a confocal stack. For example in Figure 5G the left brain lobe has a yellow cell adjacent to a green cell, but the cell is not yellow when looking at a single slice. A second similarity is that AH.lacZ ventral cord expression is seen in a subset of the lateral-most Toy cells (Figure 5G’). One aspect of larval expression that is distinct from embryonic expression is that KH.lacZ expression in the larval ventral cord looks similar to AH.lacZ (Figure 5H’) whereas KH.lacZ has no embryonic expression. This suggests that repressors of embryonic *toy* ventral cord activators present in KH.lacZ do not affect larval *toy* ventral cord activators.

A second antibody that showed coexpression with AH.lacZ was the transcription factor Drifter (also known as ventral veinless, Figure 5J,J’). Roughly symmetrical sets of 4-5 nuclei in a dorsal anterior region of the central brain near the midline express both. Topographically these nuclei appear to belong to cells of the pars intercerebralis, an important neurosecretory region that contains insulin producing cells that secrete dILP2 (Géminard *et al.* 2009). We then examined dILP2 and AH.lacZ. dILP2 is expressed in 7-9 insulin producing cells in the pars intercerebralis per brain hemisphere. Coexpression of AH.lacZ and all dILP2 cells was observed (Figure 5K,K’). Axons from the coexpressing insulin producing cells extend posteriorly and connect with a single coexpressing cell that has not yet been identified. The presence of AH.lacZ in neurosecretory cells suggests that the non-autonomous requirement for *dCORL* in EcR-B1 activation may be due to synergy between Myoglianin and insulin signaling.

## Discussion

**Figure 6:**
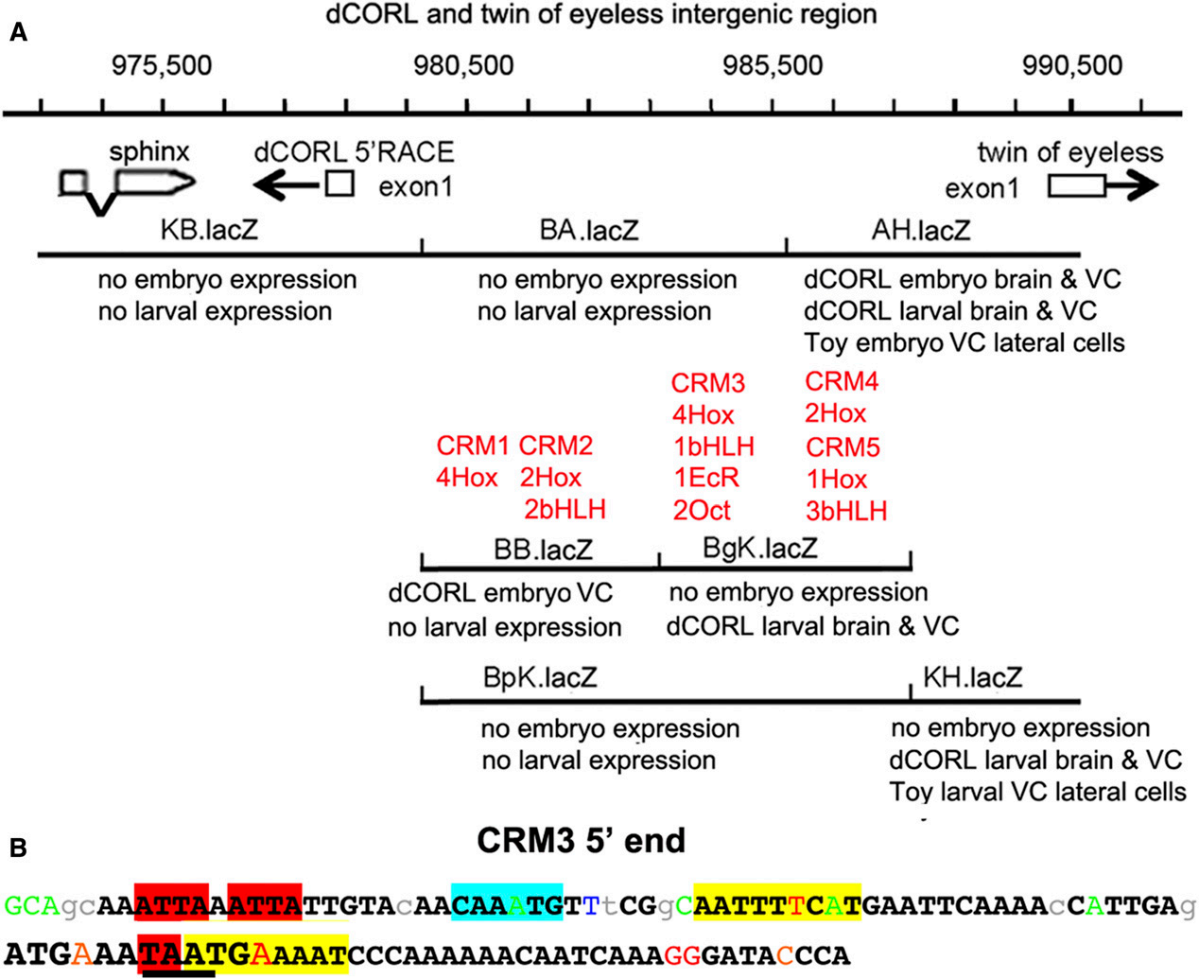
Distinct expression patterns of overlapping reporters and the location of conserved binding sites suggest the presence of intertwined activators and repressors. A) Genomic map with the addition of reporter embryonic and larval expression as well as the location of conserved sequences that may represent *cis*-regulatory modules (CRMs). The existence of interwined enhancers and repressors is illustrated in the region of overlap for AH.lacZ, BgK.lacZ and BpK.lacZ. In AH.lacZ there is *dCORL* embryonic and larval expression. However, in BgK.lacZ there is only larval expression and in BpK.lacZ there is no expression. Located in this region, CRM#4 and CRM#5 could function as activators and/or repressors of embryonic and larval expression. The juxtaposition of *dCORL* embryo expression in BB.lacZ and no expression in the overlapping BA.lacZ and BpK.lacZ, where CRM#1 and CRM#2 are located, is a another example of intertwined activators and/or repressors at either the embryonic or larval stages. CRM#3 lies in the region where BA.lacZ, BgK.lacZ and BpK.lacZ overlap with only BgK.lacZ showing any expression. CRM3# could be an activator and/or a repressor at the embryonic or the larval stage. B) Illustration of clustered and overlapping consensus transcription factor binding sites in the 5′ end of CRM#3. The 107bp nearest to *dCORL* within the CRM#3 cluster is shown. Bases in black and bold are conserved in the 12 reference Drosophila species. Lower case letters are not conserved in two or more species and colored letters are not conserved in a single species. From left to right, two core Hox binding sites are highlighted in red, a bHLH binding site in aqua, and an inverted repeat of Octamer sites (anti-parallel site upstream). The third Hox site is partially contained within the second Octamer site and illustrated with underlining. Overlapping both the third Hox site and the Octamer site is an EcR site illustrated in large font. The full CRM#3 sequence and additional details in Table S2.

Overall our reporter data shows that there are stage specific interactions between *dCORL* activators and repressors located in the intergenic region shared with *toy*. Given the complexity of *dCORL* regulation we located potential *cis*-regulatory modules (CRMs) in the intergenic region utilizing *cis*-Decoder and Evoprinter (Brody *et al.* 2007, Yavatkar *et al.* 2008). Cis-Decoder identified conserved motifs within sequence alignments generated by Evoprinter of the *dCORL* – *toy* intergenic region for twelve sequenced Drosophila species. Initially, conserved sequences were analyzed for clusters of Hox and bHLH binding sites that function as transcriptional activators (Heidt *et al.* 2007). We found five clusters of conserved Hox/bHLH sites that were then mapped to a reporter gene and assigned a role based on that reporter’s expression pattern (summarized in Figure 6A with details in Table S2).

Clusters are numbered from proximal to distal with regard to the *dCORL* transcription start site. CRM#1 and CRM#2 are close together in the region where BB.lacZ, BA.lacZ and BpK.lacZ overlap. Reporter expression suggests that CRM#1 and CRM#2 could activate or repress *dCORL* embryonic ventral cord expression. CRM#3 is located distal to CRM#2 in the region where BA.lacZ, BgK.lacZ and BpK.lacZ overlap. Reporter expression suggests CRM#3 could repress *dCORL* embryonic ventral cord or activate *dCORL* larval brain expression. CRM#4 and CRM#5 are close together and distal to CRM#3 in the region where AH.lacZ, BgK.lacZ and BpK.lacZ overlap. Reporter expression suggests CRM#4 and CRM#5 could activate or repress *dCORL* embryonic or larval brain expression. CRM#4 and CRM#5 could also activate *toy* embryonic or larval ventral cord expression. The stage specific complexity of *dCORL* regulation during embryonic and larval stages is impressive.

The bioinformatics analysis identified additional consensus motifs in the intergenic region. There are two EcR binding sites (Osz *et al.* 2015). Interestingly, one EcR site overlaps a Hox site in CRM#3 (Figure 6B) and the other overlaps a bHLH site in CRM#5 (Table S2). If the Hox/bHLH clustered CRMs serve as activators and if dCORL modulates EcR-B1 expression in the mushroom body via insulin signaling, then perhaps the EcR binding sites upstream of *dCORL* serve as repressors in a negative feedback loop antagonizing insulin signaling. This hypothesis is supported by studies of dDOR in the fat body where dDOR acts as a cofactor for EcR in a feedback loop of ecdysone inhibition of insulin signaling (Francis *et al.* 2010).

Adding to the complexity of CRM#3, we identified an inverted repeat of POU-domain Octamer binding sites in this module (Figure 6B). In flies, Octamer sites are a frequent component of CNS specific activators (23 of 27 Octamer containing sequences tested in reporter genes were CNS specific; Brody *et al.* 2012). Further, Octamer sites are often found with clustered bHLH and Hox sites where they overlap the Hox core motif. One example of Octamer/Hox overlap in a CNS specific regulatory module is the *grainyhead* neuroblast enhancer (Kuzin *et al.* 2018). This pattern is also seen in CRM#3 where one of the Octamer sites overlaps the already overlapping EcR and Hox motifs. The coincidence of three bindings sites further supports the idea that activators and repressors are intertwined.

As noted above, the coexpression of *dCORL* (AH.lacZ) and dILP2 in insulin producing cells of the larval brain suggests that *dCORL* influences EcR-B1 expression in mushroom body neurons via insulin signaling. Another mechanism required for EcR-B1 mushroom body activation employs Ftz-f1 (Boulanger *et al.* 2011). Ftz-f1 is an orphan nuclear receptor that directly activates EcR-B1 transcription in the same cells where EcR-B1 is activated by dSmad2 (Awasaki *et al.* 2011). To determine if Ftz-f1 and Smad signaling are linked via dCORL, we examined the intergenic region for Ftz-f1 and dSmad2 sites. However, none were found.

The absence of Toy expression in the reporters (except for a few cells of the embryonic and larval ventral cord) and the absence of any effect of *Df(4)dCORL* on Toy expression in the larval brain (Figure S1) suggests that *toy* activators must be downstream of its transcription start. The lack of complete *dCORL* coverage by our reporters suggests that activators of *dCORL* expression are also present far beyond the typical location. We plan to capture these by inserting a Gal4 artificial exon into the *dCORL* transcript (Nagarkar-Jaiswal *et al.* 2015).

Our *dCORL* expression data generates numerous hypotheses for our colleagues studying mouse SKOR and human Fussel proteins regarding their function in embryonic development and adults. For example, during development the coincidence of mSKOR1/2 and CNS specific orthologs of Drifter could be examined (POU3F2 and POU3F4; Hashizume *et al.* 2018). Interestingly, in humans mutations in POU3F4 cause a form of congenital X-linked hearing loss (Pollak *et al.* 2016).

In summary, our reporter gene data show that there are complex interactions between *dCORL* CNS specific activators and repressors in the intergenic region shared with *toy*. In addition, these interactions differ between embryonic and larval stages. The presence of dCO*RL* in dILP2 insulin producing cells in the larval brain suggests that *dCORL* participates in insulin signaling. The conservation of CNS-specificity for CORL family members (SKOR/Fussel) suggests that further studies of *dCORL* will suggest new hypotheses for understanding insulin signaling in mammalian development, homeostasis and disease.

## 
